# Observation of biexcitonic emission at extremely low power density in tungsten disulfide atomic layers grown on hexagonal boron nitride

**DOI:** 10.1038/s41598-017-00068-0

**Published:** 2017-03-23

**Authors:** Mitsuhiro Okada, Yuhei Miyauchi, Kazunari Matsuda, Takashi Taniguchi, Kenji Watanabe, Hisanori Shinohara, Ryo Kitaura

**Affiliations:** 1 Department of Chemistry, Nagoya University, Nagoya, 464-8602 Japan; 2 Institute of Advanced Energy, Kyoto University, Uji, Kyoto 611-0011 Japan; 3 National Institute for Materials Science, 1-1 Namiki, Tsukuba, 305-0044 Japan

## Abstract

Monolayer transition metal dichalcogenides (TMDCs) including WS_2_, MoS_2_, WSe_2_ and WS_2_, are two-dimensional semiconductors with direct bandgap, providing an excellent field for exploration of many-body effects in 2-dimensions (2D) through optical measurements. To fully explore the physics of TMDCs, the prerequisite is preparation of high-quality samples to observe their intrinsic properties. For this purpose, we have focused on high-quality samples, WS_2_ grown by chemical vapor deposition method with hexagonal boron nitride as substrates. We observed sharp exciton emissions, whose linewidth is typically 22~23 meV, in photoluminescence spectra at room temperature, which result clearly demonstrates the high-quality of the current samples. We found that biexcitons formed with extremely low-excitation power (240 W/cm^2^) at 80 K, and this should originate from the minimal amount of localization centers in the present high-quality samples. The results clearly demonstrate that the present samples can provide an excellent field, where one can observe various excitonic states, offering possibility of exploring optical physics in 2D and finding new condensates.

## Introduction

Two-dimensional (2D) transition metal dichalcogenides (TMDCs) provide a fascinating playground for studies of optical and device physics in two dimensions. TMDCs including MoS_2_, WS_2_, MoSe_2_ and WSe_2_ are layered semiconductors that are composed of a transition metal layer sandwiched by chalcogen layers with trigonal prismatic coordination geometry^1^. Importantly, these TMDCs can be isolated as their monolayer form, and they are 2D direct-gap semiconductors at monolayer thickness^2–4^. Due to their 2D structure, many-body effects in TMDCs are prominent, which, for example, results in strong excitonic effects in optical transitions^5^. In fact, photoluminescence (PL) spectra of monolayer TMDCs exhibit strong emissions arising from excitons (electron-hole pairs) and trions (charged excitons) even at room temperature^6–8^, and these results suggest an opportunity to find further peculiar complex many-body states such as higher-order excitons, electron-hole plasma and exciton condensates in TMDCs^9–12^.

To further explore the fundamental properties of TMDCs, the important point is preparation of high-quality samples to ensure their intrinsic properties. High-quality samples can show sharp excitonic PL that arises from the long dephasing time and the minimal amount of structural roughness. This leads to easier observation of fine structures in a PL spectrum, giving more chance to observe new excitonic states^13^. In order to realize high-quality samples, we have focused on monolayer WS_2_ directly grown on hexagonal boron nitride (*h*BN)^14^, where an ideal atomically flat surface virtually without dangling bonds is available. The monolayer WS_2_ grown on *h*BN (WS_2_/*h*BN) is thus atomically flat with clean interfaces, showing PL peaks whose full-width at half-maximum (FWHM) are much smaller than those of mechanically exfoliated and samples grown onto other substrates^14–16^. Using WS_2_/*h*BN, we have observed the formation of biexcitons at 80 K even with extremely low excitation power of 2.8 μW or 240 W/cm^2^, which is 18 or 125 times smaller, respectively, than those reported previously^17, 18^.

## Results

### CVD Growth of WS_2_ on *h*BN and characterization

**Figure 1:**
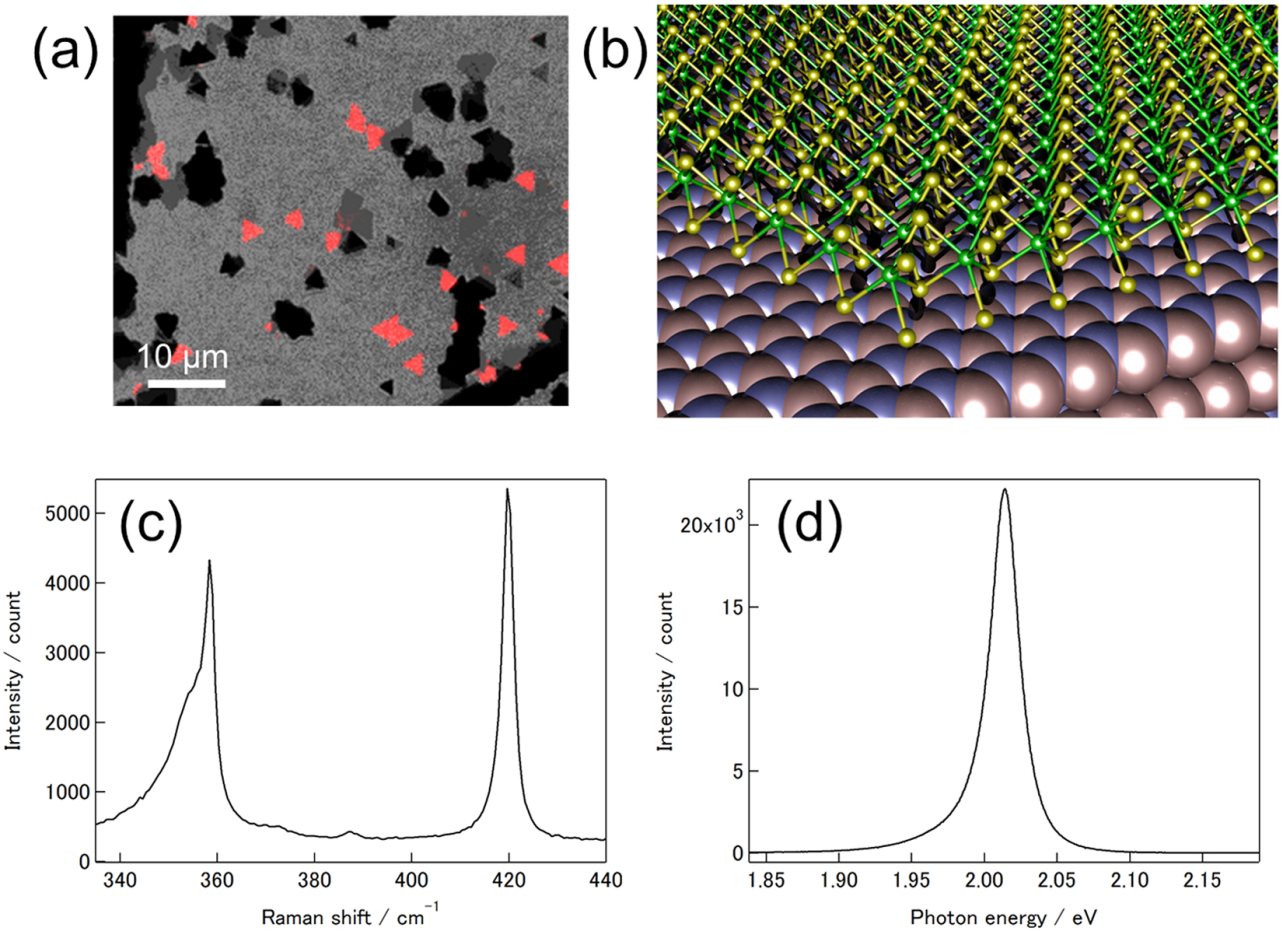
(**a**) A PL image of WS_2_/*h*BN. The PL image was taken with 488 nm excitation. This image is combination of transmission bright field image (monochrome) and 600–700 nm detection (red). (**b**) Structure model of WS_2_ grown on *h*BN. Yellow, Green, blue and orange spheres represent sulfur, tungsten, nitrogen and boron atoms, respectively. B and N atoms are drawn with van der Waals radii, and Mo and S are drawn with the ball-and-stick model. (**c**) A typical Raman spectrum of WS_2_/*h*BN. (**d**) A typical PL spectrum of WS_2_/*h*BN. The excitation energy of 2.54 eV was used to measure the Raman and PL spectrum.

We have prepared WS_2_/*h*BN by the chemical vapor deposition (CVD) method with WO_2.9_ and elemental sulfur as source materials together with exfoliated *h*BN flakes as substrates. To check the layer number of WS_2_ grown on *h*BN, we performed PL imaging, atomic force microscopy (AFM), Raman and PL spectroscopy for the respective samples. Figure 1a,b,c and d show a typical PL image, a structure model of WS_2_/*h*BN, Raman and PL spectra, respectively. Because monolayer WS_2_ is a direct-gap semiconductor, crystals of monolayer WS_2_ can be seen as bright triangular contrasts in the PL images; the triangular shape arises from preferential appearance of zig-zag edges during the growth, being consistent with previous reports on CVD-growths of TMDCs^17, 19, 20^. A typical AFM image of the WS_2_/*h*BN clearly demonstrate the triangular crystal shape with a thickness of 0.7 nm, which is consistent with the monolayer WS_2_ structure (Figure S1)^20^. The monolayer structure of WS_2_ can further be confirmed by the Raman spectrum (Fig. 1c), where the peak separation between *E*′ (358.6 cm^−1^) and *A*′_1_ (419.8 cm^−1^) modes is 61.2 cm^−1^ in consistent with monolayer WS_2_^19, 21^. The strong and narrow emission peak at 2.014 eV in the PL spectrum (Fig. 1d, taken with excitation energy of 2.54 eV) can be assigned to neutral excitons of monolayer WS_2_. All results shown above clearly demonstrate the successful growth of monolayer WS_2_ on *h*BN.

Crystal quality can be confirmed by the Raman and PL spectrum. The Raman spectrum shows two strong Raman bands that are assigned to *E*′ and *A*′_1_ modes. The observed Raman bands are sharp (i.e., FWHM of the *A*′_1_ Raman band is 2.8 cm^−1^)^17, 22^, which clearly demonstrates the high crystallinity of the current WS_2_ crystal. The observed FWHM of the PL emission in Fig. 1c, 22.5 meV, is much smaller than those of the exfoliated WS_2_ and the CVD-grown WS_2_ (grown on SiO_2_/Si or sapphire substrates); typical values of FWHM reported range from 42 to 75 meV^19, 23, 24^. The small FWHM should originate from high quality of the grown WS_2_/*h*BN, indicating less surface roughness and amount of defects. The less surface roughness can be directly confirmed by the height profile of the AFM (Figure S1), where the atomically flat surface of WS_2_ grown on *h*BN is clearly seen. A transmission electron microscopy (TEM) image of WS_2_/*h*BN (shown in Figure S2) shows lattice fringes of WS_2_, and the corresponding electron diffraction pattern clearly demonstrates the match in the crystallographic orientation between WS_2_ and *h*BN, manifesting a smooth and clean interface existing between WS_2_ and *h*BN.

### Temperature dependence in photoluminescence of WS_2_/*h*BN

**Figure 2:**
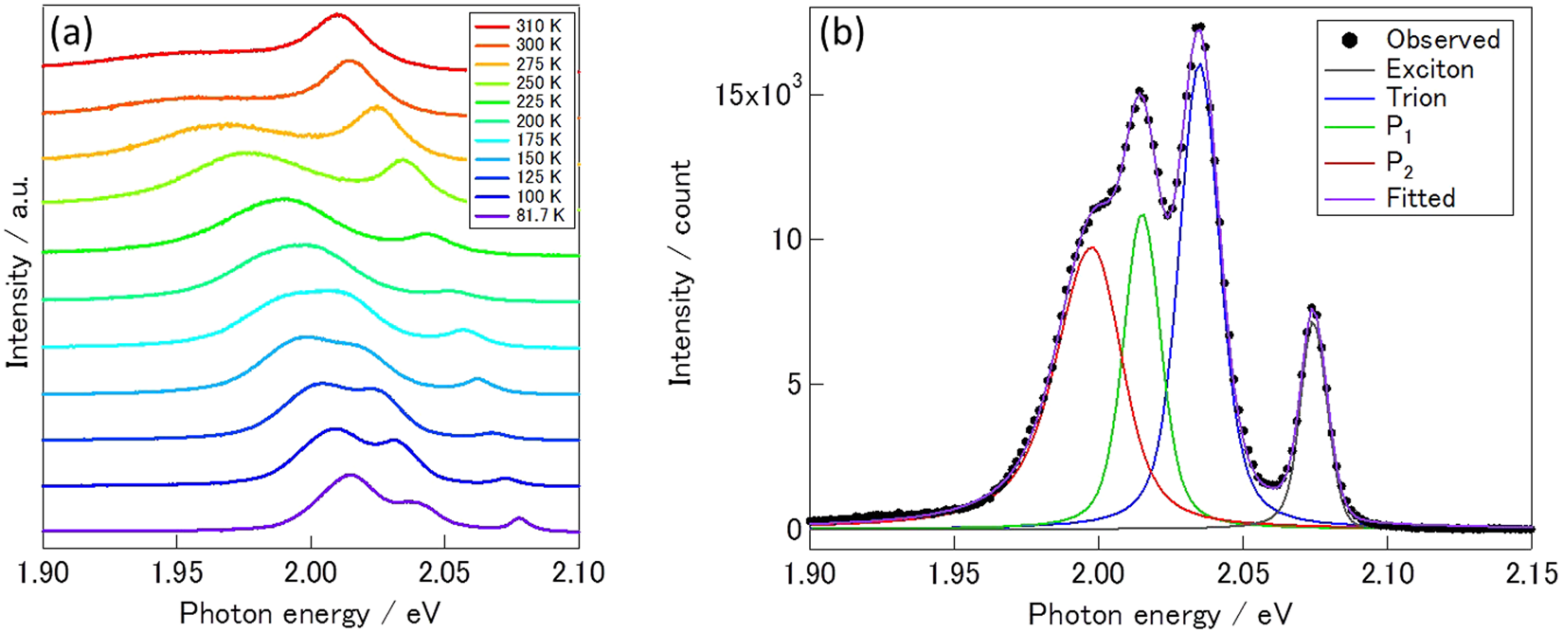
(**a**) PL spectra of WS_2_/*h*BN measured at temperatures ranging from 81.7 to 310 K. (**b**) A PL spectrum of WS_2_/*h*BN measured at 80 K. Contribution from excitons, trions, P_1_ and P_2_, which are shown by black, blue green and red curves, respectively, are modeled by the pseudo-voigt function. Excitation energies of 2.54 eV and 2.38 eV were used to measure spectra in (**a**) and the spectrum in (**b**), respectively.

To further explore excitonic states in WS_2_, we measured temperature dependence of PL spectra. Figure 2a shows PL spectra of WS_2_/*h*BN measured at temperatures ranging from 81.7 to 310 K. As seen in Fig. 2a, the emission peak from excitons shifts to the lower energy side when temperature decreases. The peak shift can be well fitted by Varshni’s formula (Figure S3 and Table S1), which explains bandgap change caused by electron-phonon interaction^25^. As seen in Fig. 2a, additional peaks appear when temperature is lower than 225 K. We used excitation energy of 2.54 eV to measure the PL spectra, and electrons and holes generated by the excitation relax to the conduction band bottom as well as the valence band top at K (K′) points in Brillouin zone, leading to formation of neutral free excitons^26^. At low temperature, fee excitons generated can be trapped by localized centers or can form trions, while these states are not observable at high temperature due to the thermal dissociation into free excitons. In the case of WS_2_ grown on sapphire, no additional PL peaks are observed in PL spectra even at 80 K, which fact clearly shows significance of the substrate effect to observe fine structures of excitonic states (Figure S4).

Figure 2b shows the PL spectrum of WS_2_/*h*BN at 80 K, where three additional emission peaks are seen at the lower energy side of the PL of neutral excitons. As clearly seen in the spectrum, the emission peaks are well-separated each other, leading to an easy identification of additional excitonic states in the present sample. The emission at 2.036 eV can be assigned to trions based on the binding energy of 39 meV, and the formation of trions in the present sample indicates that there are accidentally-doped free carriers in the sample^27^. The intensity of the trion emission at room temperature is almost negligible, which results from small amount of the free carriers in the sample.

**Figure 3:**
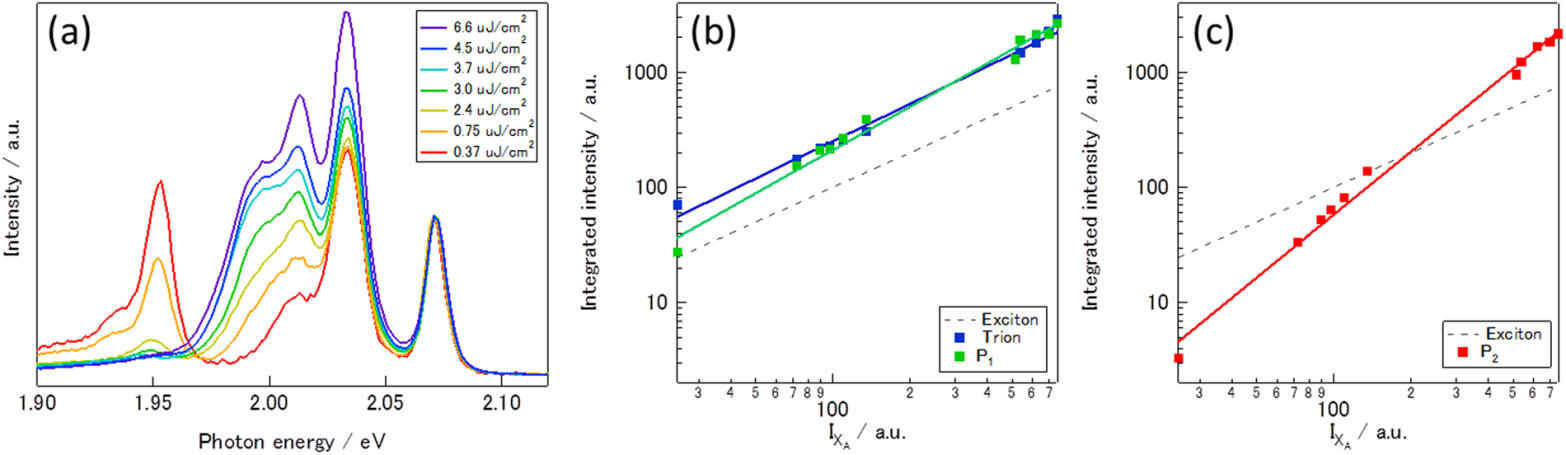
(**a**) PL spectra of WS_2_/*h*BN measured with excitation power ranging from 0.37 to 6.6 μJ/cm^2^ at 80 K. (**b,c**) Plots of PL intensities of trion, P_1_ and P_2_ verses that of exciton. Green, blue and red filled squares correspond to PL intensities of P_1_, trion and P_2_, respectively. Solid lines corresponds to fitted lines based on the relation of *I* ∞ *I*_ex_^*α*^.

In addition to emission peaks from neutral excitons and trions, we can see two strong emission peaks at 2.015 (P_1_) and 1.998 (P_2_) eV. To address the origin of the additional peaks, we measured excitation power dependence of PL spectra. In this measurements, we used excitation energy of 2.38 eV with pulse width of 20 ps. Figure 3a shows PL spectra measured with the excitation power ranging from 0.37 to 6.6 μJ/cm^2^ at 80 K; the intensities of the PL spectra are normalized in such a way that intensity of neutral exciton is one. As clearly seen in the figure, P_2_ peak becomes prominent as excitation power increases, whereas P_1_ peak is almost comparable to the neutral exciton peak throughout the all spectra.

## Discussion

Figures 3b and c show the relation in PL intensity between the exciton emission and other emissions. As expected, the intensity of the trion emission is almost proportional to that of the neutral exciton emission; a fitting using the equation, *I*_tri_ ∝ *I*_ex_^*α*^ (*I*_tri_ *, I*_ex_ and *α* correspond to the intensity of trion emission, the intensity of exciton emission and constant, respectively) gives *α* of 1.08. The intensity of P_1_ is also almost proportional to that of exciton, and a fitting using the same equation yields *α* of 1.25. The obtained value of *α*, which is close to 1, means that the P_1_ state is also formed through a one-exciton process where an exciton, for example, captures a free carrier, namely a fine structure of trion, or is trapped by impurities.

In contrast, in Fig. 3c, the intensity of P_2_ clearly shows super-liner relation against the intensity of excitons, providing *α* value of 1.82. The observed super-liner relation strongly suggests that the P_2_ emission peak originates from biexcitons^28, 29^. The observed binding energy of the P_2_ peak is ca. 75 meV, which yields Haynes factor (the ratio in binding energy between excitons and biexcitons) of 0.09–0.23; the exciton binding energy reported ranges from 0.32 to 0.71 eV^7, 8, 30^. The calculated Haynes factor is consistent with those of quantum-well systems and other TMDCs^28, 31, 32^. Valley polarization measurements (Figure S6) also support that the P_2_ peak originates from biexcitons. As shown in Figure S6, P_2_ peak shows a valley polarization of 12.7%, which excludes the possibility that the P_2_ peak originates from bound excitons or bound biexcitons^28^.

**Figure 4:**
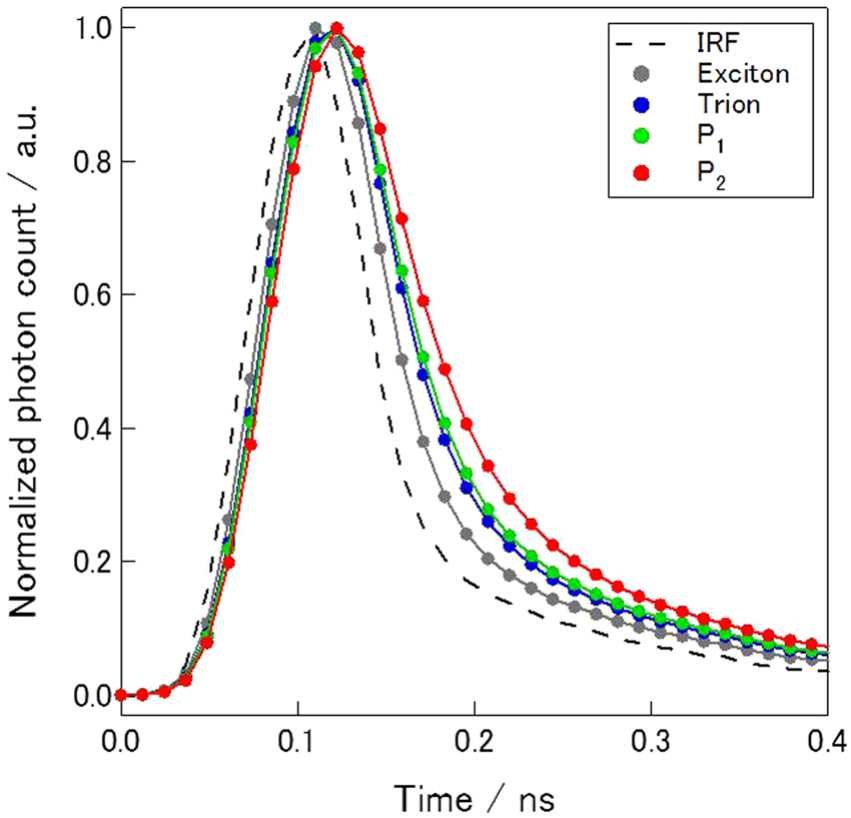
A time dependence in PL intensities of excitons, trions, P_1_ and P_2_ of WS_2_/*h*BN. Dashed line corresponds to the impulse response function of experimental setup used to measure the time dependence in PL intensities.

Figure 4 shows time-resolved PL intensities. Fitting of the experimental curves in Fig. 4 based on a single exponential decay yielded the relaxation time of excitons (*τ*_ex_), trions (*τ*_tri_) and biexcitons (*τ*_b_) as 15, 22 and 31 ps, respectively. When thermal equilibrium between excitons and biexcitons is reached, *τ*_ex_ should be twice as large as *τ*_b_. This is, however, not the present case because the measurement was performed at 80 K and the biexciton binding energy of 75 meV is much larger than the thermal energy of 80 K (6.9 meV), which situation should make the biexciton to exciton conversion extremely slow. The temperature, 80 K, is almost ten times smaller than the binding energy of biexcitons, leading to the complete blocking of the biexciton to exciton conversion process^28, 33^. The observed relation between *τ*_X_ and *τ*_XX_ is therefore consistent with the formation of biexcitons with the large binding energy (for details, see Supporting information)^28^. The observed binding energy of biexciton, 75 meV, is larger than those reported previously^17, 18, 34, 35^. Biexcitons are larger than excitons, and biexcitons are supposed to be more sensitive to the screening. The different environment and density of free carrier in WS_2_/*h*BN might contribute to the observed large binding energy.

**Figure 5:**
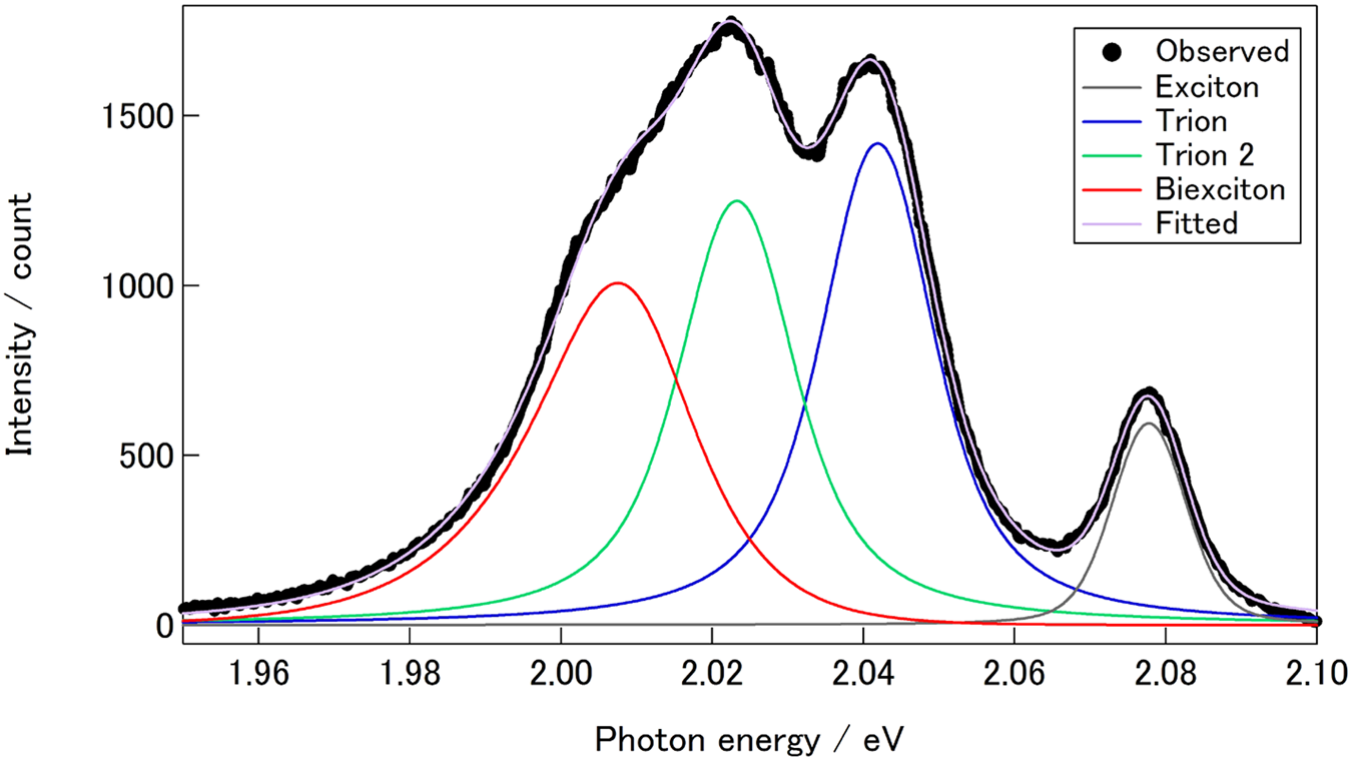
A PL spectrum of WS_2_/*h*BN measured with low excitation power, 2.8 μW/240 W/cm^2^. Solid lines correspond to contributions from excitons, trions, P_1_ and P_2_ peaks, which were modeled by the pseudo-voigt function. The excitation energy of 2.54 eV was used to measure the spectrum.

In previous studies, the biexciton emission have been observed with excitation power of, for example, 50 μW and 30 kW/cm^2^ at 77 and 4 K, respectively^17, 18^. In contrast, in our samples, the biexciton emission can be clearly seen with much smaller excitation power of 2.8 μW or 240 W/cm^2^ at 81.7 K (Fig. 5). Considering the absorption coefficient reported^20^, the excitation power can be converted to the maximum density of excitons generated, 2.5 × 10^8^/cm^2^, giving the average exciton-exciton distance as 630 nm. In this experiment, biexcitons were formed through the exciton-exciton collision, and the exciton-exciton distance should be comparable to the exciton diffusion length, *L*_*ex*_. The *L*_*ex*_ can be calculated using the following formula, *L*_*ex*_ = 2(*D*_ex_ *τ*_*ex*_)^1/2^, where *τ*_*ex*_ and *D*_ex_ are the lifetime (15 ps) and the diffusion coefficient of exciton, respectively. The exciton diffusion coefficient can be estimated from the Einstein relation *D*_ex_ ~ *k*_*B*_ *T*/*M*_*ex*_ *Δ*, where *k*_*B*_, *M*_*ex*_ and *Δ* are the Boltzmann constant, the exciton translational mass (~0.55 *m*_*0*_)^36^ and homogeneous linewidth of exciton (~3.2 meV); the homogeneous linewidth was determined through spectral fitting with the Voigt function. Using the obtained *D*_ex_ of ~9.3 cm^2^/s, *L*_*ex*_ was estimated to be ~240 nm, which is comparable to the average exciton-exciton distance. The small homogenous linewidth of the exciton emission means that the dephasing time of excitons in the present sample is long, leading to the long *L*_*ex*_ up to 240 nm even with the short *τ*_*ex*_. The long *L*_*ex*_ might originate from significant decrease in roughness scattering in ultraflat WS_2_ in our WS_2_/*h*BN samples.

In addition to the flatness, the small amount of trap centers and free carriers in WS_2_/*h*BN also helps to form biexcitons. As shown in Fig. 3a, PL emission from localized excitons, which have been observed typically at 1.8~2.0 eV, is strongly suppressed under higher excitation power^34^. This means that the amount of the trap centers in the current samples is smaller than those in previous studies^34^. In addition, as already discussed, the amount of free carriers in the present sample is small, which lead to less formation of trions. Higher order excitons including biexcitons are formed through collisions of excitons, and other processes such as the formation of localized excitons and trions compete with the formation of biexcitons. The fewer amount of trap centers thus contribute to an easy observation of biexcitons in our high-quality samples. The difference may originates from the difference in substrates, and surface roughness, charged impurities and dangling bonds in ordinal substrates like SiO_2_/Si are the origin of the trap centers^37^.

## Conclusion

In conclusion, we have grown a high-quality WS_2_ on *h*BN substrates by the CVD method. The present WS_2_/*h*BN sample shows a sharp PL emission at room temperature, and the low temperature PL shows additional peaks originating from biexcitons. The power dependence and valley polarization measurements are consistent with the formation of biexcitons. Biexcitons are formed with extremely low-excitation power, 240 W/cm^2^, which originates from the minimal amount of localization centers in the present high-quality samples. These results clearly demonstrate that the present samples can provide an excellent field, where one can observe various excitonic states, offering possibility of exploring optical physics in 2D and finding new condensates.

## Methods

### CVD growth of WS_2_ on *h*BN

We have grown WS_2_ atomic layers by CVD method with elemental sulfur (Sigma-Aldrich, 99.998%) and tungsten oxide (WO_2.9_, Alfa Aesar, 99.99%) as precursors. We prepared thin *h*BN flakes, whose thickness is typically several tens of nanometers, on sapphire or quartz substrate by the mechanical exfoliation method, and the exfoliated thin *h*BN flakes were used as substrates for the CVD growth of WS_2_ atomic layers. A substrate with *h*BN flakes, WO_2.9_ and sulfur were placed in a quartz tube with diameter of 26 mm; the WO_2.9_ was placed in a quartz tube whose inner diameter is 8 mm to avoid rapid sulfurization of WO_2.9_. Under Ar flow of 200 sccm, the quartz tube was heated with three-zone furnace at 190, 400 and 1050 degree for 30 minutes; a substrate with *h*BN flakes and sulfur were placed at the hottest and coldest zone, respectively.

### Photoluminescence (PL) and Raman measurement

We obtained PL images by a fluorescence microscope (Leica TCS SP8 gSTED) with excitation wavelength of 488 nm at room temperature. Raman and PL spectra were measured by using a confocal-microscope based Raman microscopy (Jobin Yvon LabRAM HR-800) with 488 nm excitation (CW laser, COHERENT Sapphire 488 LP). In measurements of Raman spectra, a notch filter was used to filter out the intense signal from Rayleigh scattering. An objective lens (100 x, 0.9 NA) was used to focus the laser light onto a sample and collect the backscattered light from the sample. Raman and PL signal were detected with a charge-coupled device. In temperature dependence measurements, we placed WS_2_/*h*BN in a cryostat (CryoVac KONTI-Cryostat-Micro) with continuous flowing of liquid N_2_ under vacuum of ~10^−6^ Torr. Temperature was controlled by a CryoVac TIC 304-MA.

### AFM and TEM observations

AFM images were obtained with a Veeco Dimension 3100. High-resolution TEM images were taken with a JEM-2100F (JEOL) operated at 80 keV. We transferred *h*BN flakes with grown WS_2_ onto a copper TEM grid by the standard polymer-based transfer technique. TEM images were recorded with a charge-coupled device with exposure time of typically 1~3 seconds. Electron energy loss spectroscopy (EELS) and energy-dispersive X-ray (EDX) spectroscopy were carried out with a post column spectrometer (gatan Enfina) and an in column spectrometer, respectively.

## Electronic supplementary material

Supplementary information
