# Supplementary material for: Observation of biexcitonic emission at extremely low power density in tungsten disulfide atomic layers grown on hexagonal boron nitride
Source: Sci Rep. 2017 Mar 23;7:322. doi: 10.1038/s41598-017-00068-0 (PMC5428332; doi:10.1038/s41598-017-00068-0)
Supplement: Supplementary file 1 — Supplementary information [file 41598_2017_68_MOESM1_ESM.docx]

Supporting Information

Observation of biexcitonic emission at extremely low power density in tungsten disulfide atomic layers grown on hexagonal boron nitride

Mitsuhiro Okada^1^, Yuhei Miyauchi^2^, Kazunari Matsuda^2^, Takashi Taniguchi^3^, Kenji Watanabe^3^ and Hisanori Shinohara^1,^* and Ryo Kitaura^1,^*

*^1^Department of Chemistry, Nagoya University, Nagoya 464-8602 Japan*

*^2^Institute of Advanced Energy, Kyoto University, Uji, Kyoto 611-0011, Japan*

*^3^National Institute for Materials Science, 1-1 Namiki, Tsukuba, 305-0044, Japan*

**Surface flatness of WS_2_/hBN.**

Figure S1 shows an AFM image and the corresponding height profile of a WS_2_/hBN. As seen in the figures, there is a WS_2_ crystal with triangular shape, whose height is about 0.8 nm. Surface of WS_2_ is atomically flat, which can clearly be seen in the height profile; surface roughness of WS_2_ on hBN is within the rage of 0.1 nm.


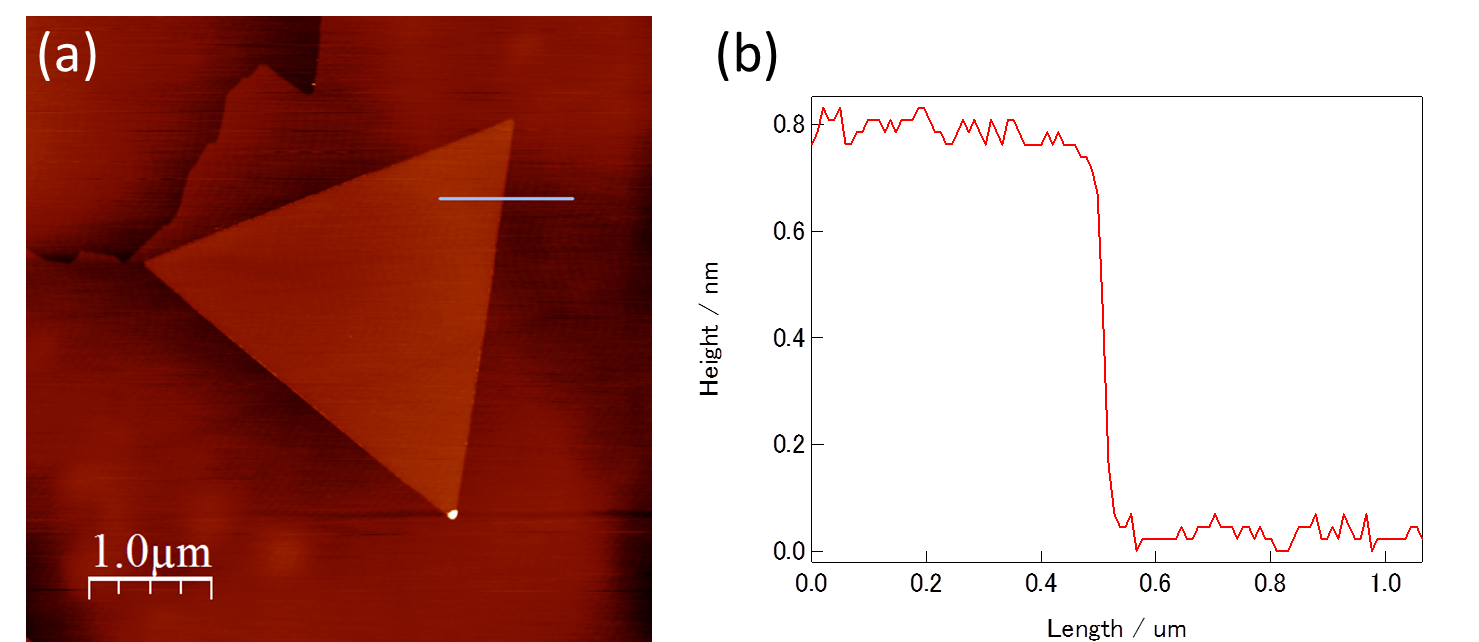


Fig. S1 (a) an AFM height image. (b) a height profile along the blue line shown in Fig. S1(a).

**Structural characterization with TEM**

Figure S2(a) shows a TEM image of WS_2_/hBN, where lattice fringes are seen. The corresponding electron diffraction pattern shown in Fig. S2(b) shows two sets of hexagonal patterns, which originates from WS_2_ and hBN with hexagonal crystal system. The orientations of the two hexagonal patterns match well, and this means that WS_2_ formed through the van der Waals epitaxial growth mode. Elemental analyses with EELS and EDX clearly show the existence of boron, nitrogen, sulfur and tungsten atoms, which is consistent to WS_2_/hBN. Signal from Cu in the EDX spectrum originates from a copper TEM sample grid.


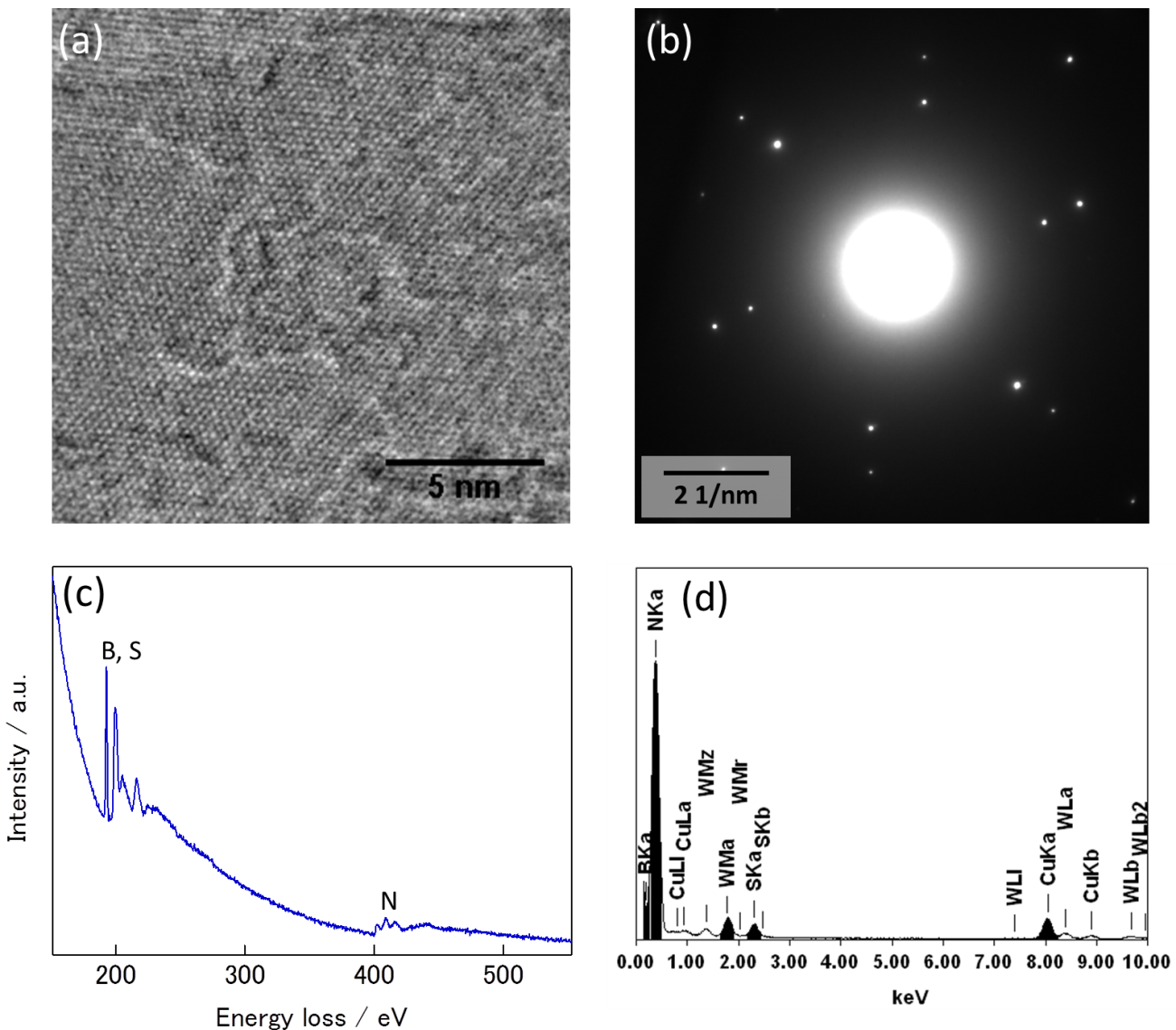


Fig.S2 (a) a typical TEM image of WS_2_/hBN. (b) an electron diffraction pattern of WS_2_/hBN. (c) EELS (Electron Energy Loss Spectrometry) spectrum of WS_2_/hBN. (d) EDX (Energy Dispersive X-Ray Spectrometry) spectrum of WS_2_/hBN.

**Varshini’s plot**

Figure S3 shows temperature dependence of PL peak positions of exciton, trion and P_1_. All peaks show blue shift with decreasing temperature, which are fitted by Varshini’s equation shown below.

$$E_{g}\left( T \right)=E_{g}\left( 0 \right)-\frac{\alpha T^{2}}{T+\beta}$$

*a* and *b* are fitting parameters, *T* is temperature and *E_g_*(0) is the bandgap at *T* = 0. Varshini’s equation is an empirical relation, which have been widely used to represent temperature dependence of bandgap. All temperature dependences in the peak positions are fitted well with the fitting parameters listed in Table S1. This means that the observed temperature dependence originates from change in bandgap of WS_2_.

Figure S3 Gray, blue and green squares correspond to peak positions of exciton, trion and P_1_, respectively. Solid lines represent fitting curves based on Varshini’s equation.

Table S1 : Varshni’s parameter

|  |  | Obtained | values |  |
| --- | --- | --- | --- | --- |
| Exciton | α / eVK^-1^ | 0.000589 | ± | 7.99×10^-6^ |
|  | β / K | 514.07 | ± | 10.6 |
|  | E(0) / eV | 2.0825 | ± | 0.000536 |
| Trion | α / eVK^-1^ | 0.000719 | ± | 9.36×10^-6^ |
|  | β / K | 480.04 | ± | 9.69 |
|  | E(0) / eV | 2.0445 | ± | 0.000655 |
| P_1_ | α / eVK^-1^ | 0.000657 | ± | 0.000016 |
|  | β / K | 473.47 | ± | 16.2 |
|  | E(0) / eV | 2.0207 | ± | 0.000712 |

**Temperature dependence PL spectra of WS_2_ grown on Sapphire (0001)**

Figure S4 (a) shows temperature PL spectra measured at temperatures ranging from 81.7 to 310 K. The blue shift with decreasing temperature originates from temperature dependent bandgap change, which is also observed in WS_2_ grown on hBN. Figure S4 (b) shows a PL spectrum measured at 81.7 K. Although a shoulder is seen at around 2 eV, the new features observed in WS_2_/hBN cannot be seen clearly.


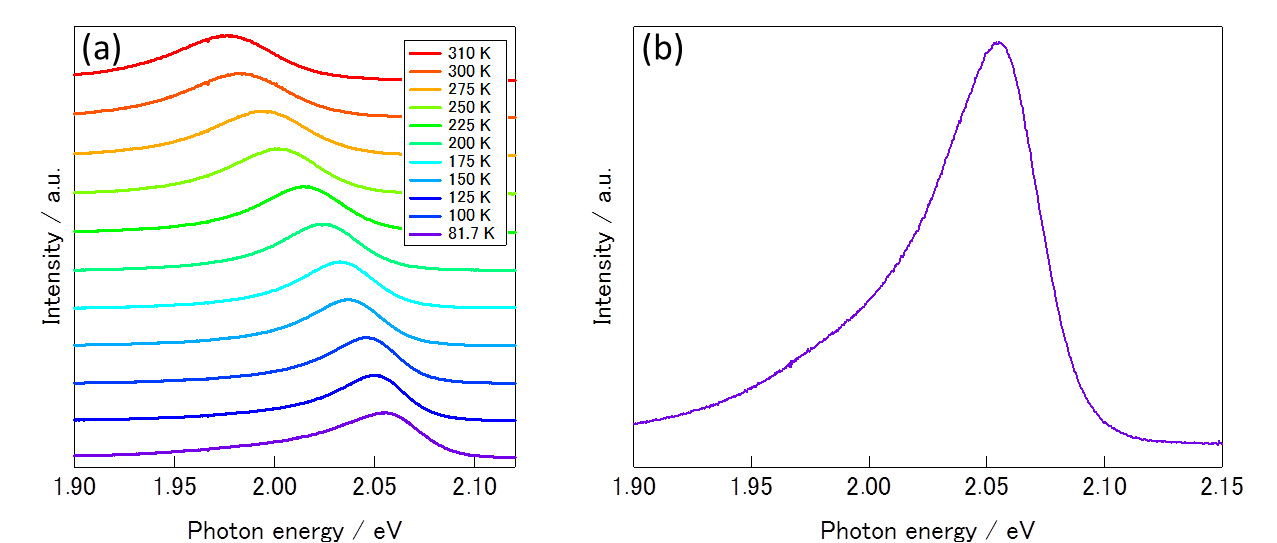


Figure S4 Temperature dependence in PL spectra of WS_2_ grown directly on sapphire (0001) surface. (a) PL spectra measured at temperatures ranging from 81.7 K to 310 K. (b) PL spectra measured at 81.7 K.

**An another example of excitation power dependence of PL spectra of WS_2_/hBN**

Figures S5 show excitation power dependence in PL spectrum and PL intensity of trion, P_1_ and P_2_.


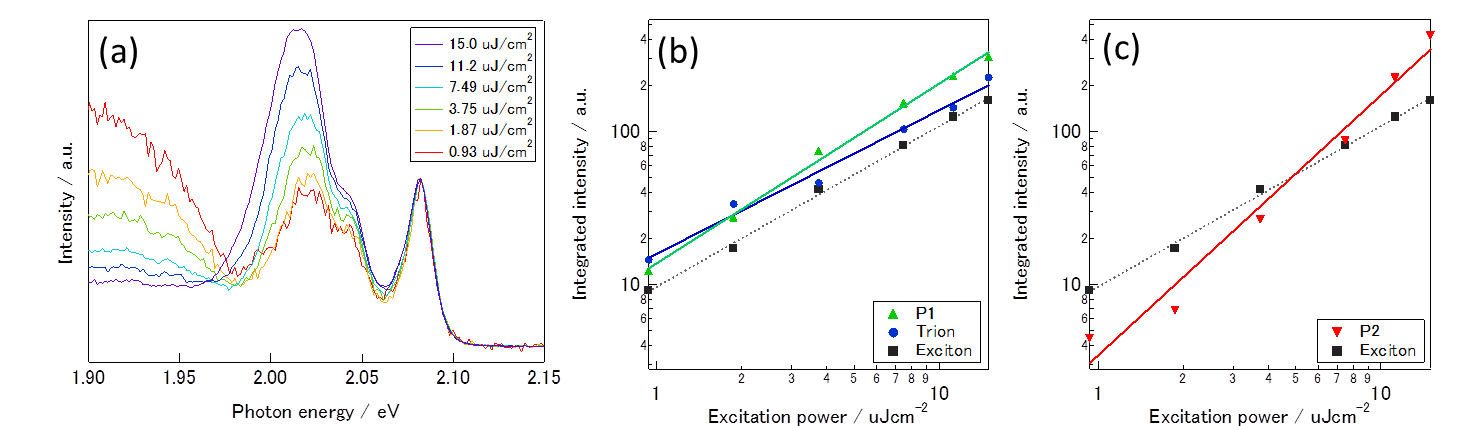


Figure S5 (a) Excitation power dependence of PL spectra of WS_2_/hBN. (b), (c) Excitation power dependence in PL intensity of trion, P_1_ and P_2_.

**Valley polarization of WS_2_/hBN**

Fig. S6 shows PL spectra measured with circularly polarized excitation at 54 K. As clearly seen, there is difference in PL intensity measured with different polarization, σ+ and σ-. The difference exists not only in exciton and trion but also in P_1_ and P_2_ peaks, which difference means that valley polarization exists in all contributions. The valley polarizations calculated based on the PL spectra are shown in Table S2. The existence of valley polarization excludes possibility that P_1_ and P_2_ arise from localized states.

Figure S6 PL spectra measured with circularly polarized excitation. We use excitation energy of 2.33 eV and 15 *µJ*/*cm*^2^ to measure the PL spectra.

Table S2 : Valley polarization of each peak

| State | Exciton | Trion | P1 | P2 |
| --- | --- | --- | --- | --- |
| Polarization | 0.176 | 0.266 | 0.201 | 0.127 |

The valley polarizations were calculated from the following equation:

$$P=\frac{I\left( \sigma+ \right)-I\left( \sigma- \right)}{I\left( \sigma+ \right)+I\left( \sigma- \right)}$$

**On the lifetime of biexcitons.**

Assuming the following processes,

$$X+X\rightleftharpoons XX$$

$$X\to X+T$$

$$XX\to X+\hbar\omega_{\mathrm{XX}}$$

$$X\to\hbar\omega_{X}$$

where X, XX, T, $\omega_{X}$ and $\omega_{\mathrm{XX}}$ represent exciton, biexciton, trion, frequency of biexciton emission and frequency of exciton emission, the rate equations on number of excitons and biexcitons are the followings.

${\frac{d}{dt}n}_{X}\left( t \right)=G-\frac{2}{\tau_{\mathrm{EB}}}{n_{X}}^{2}-\frac{1}{\tau_{X}}n_{X}-\frac{1}{\tau_{\mathrm{ET}}}n_{X}-\frac{1}{\tau_{\mathrm{NR}}}n_{X}$ (1)

${\frac{d}{dt}n}_{XX}\left( t \right)=\frac{2}{\tau_{\mathrm{EB}}}{n_{X}}^{2}-\frac{1}{\tau_{\mathrm{BE}}}n_{\mathrm{XX}}-\frac{1}{\tau_{\mathrm{XX}}}n_{\mathrm{XX}}-\frac{1}{\tau_{\mathrm{XXNR}}}n_{\mathrm{XX}}$ (2)

The first term in equation (1), G, corresponds to exciton generation function, and *t*, *n*_X_ and *n*_XX_ represent time, the number of excitons and biexcitons. *τ*_EB_, *τ*_BE,_ *τ*_x_, *τ*_ET_, *τ*_NR_, *τ*_XX_ and *τ*_XXNR_ represent lifetimes of exciton-biexciton conversion, biexciton-exciton conversion, exciton radiative decay, exciton-trion conversion, exciton nonradiative decay, biexciton radiative decay and biexciton nonradiative decay. The simultaneous differential equations, (1) and (2), cannot be solved analytically, but assuming single exponential decay in the number of exciton,

$n_{X}\left( t \right)=n_{X}\left( 0 \right)\exp\left( -\frac{t}{\tau_{X,obs}} \right)$ **,**

the analytical expression of the number of biexcitons can be derived. *n*_x_(0) and *τ*_x,obs_ correspond to the number of exciton at *t* = 0 and the life time of exciton decay. The analytical expression of the number of biexcitons is the followings.

$n_{\mathrm{XX}}\left( t \right)=\frac{\tau_{1}\tau_{2}}{\tau_{1}-\tau_{2}}\frac{n_{X}\left( 0 \right)}{\tau_{\mathrm{EB}}}\left( \exp\left( -\frac{t}{\tau_{1}} \right)-\exp\left( -\frac{t}{\tau_{2}} \right) \right)$ (3)

$$\frac{1}{\tau_{1}}=\frac{2}{\tau_{X,obs}}$$

$$\frac{1}{\tau_{2}}=\frac{1}{\tau_{\mathrm{BE}}}+\frac{1}{\tau_{\mathrm{XX}}}+\frac{1}{\tau_{\mathrm{XXNR}}}$$

As shown in the equation (3), the number of biexcitons is composed of two terms. When the first term, exp(-*t*/*τ*_1_), is dominant, the lifetime of biexciton should be half of that of exciton. When the contribution from the second term is not negligible, the lifetime of biexcitons is not half of that of exciton any more. In normal quantum wells, the second term is negligible due to fast biexciton-exciton conversion, giving the lifetime of biexciton that is half of that of exciton. On the other hand, the second term contributes significantly in WS_2_/hBN due to the slow biexciton-exciton conversion arising from the large binding energy, and this makes the observed long lifetime possible.
